# The Structure of Pandemic Vulnerability: Housing Wealth, Residential Segregation, and COVID‑19 Mortality

**DOI:** 10.1007/s11113-023-09826-7

**Published:** 2023-10-09

**Authors:** Chinyere O. Agbai

**Affiliations:** 1 Department of Sociology, The Ohio State University, 238 Townshend Hall, 1885 Neil Ave Mall, Columbus, OH 43210, USA

**Keywords:** COVID-19, Wealth, Housing, Segregation, Racial inequality, Social determinants of health

## Abstract

The COVID-19 pandemic has been particularly devastating for those with limited economic resources. Extensive research demonstrates the negative relationship between wealth and mortality at both the individual and area levels. In addition, residential segregation has been linked to poor health and greater mortality. Home equity is the largest asset that many Americans own, but residential segregation devalues homes located in Black neighborhoods. Despite the interlocking relationships between wealth, residential segregation, and mortality, it remains unclear how wealth and residential segregation work to predict COVID-19 deaths. Using U.S. Census data and county-level COVID-19 data from Johns Hopkins University (*n* = 1164), I deploy median home value as a wealth proxy and negative binomial regression models to interrogate two questions. (1) What is the relationship between home value and COVID-19 deaths? (2) How does the relationship vary by level of residential segregation? Results indicate that COVID-19 mortality is 64 percent greater in the lowest wealth counties than in the wealthiest counties. At average median home value, the most segregated counties with the largest Black populations suffer 28 percent more COVID-19 deaths than similarly situated counties with low levels of residential segregation and small Black populations. This study underscores the importance of accounting for residential segregation in examinations of the well-established relationship between socioeconomic status and health and mortality.

## Introduction

The ongoing COVID-19 pandemic highlights numerous existing dimensions of social inequality. Those with greater wealth have been able to retreat to second homes in more rural areas to avoid the spread of the virus in densely populated cities. The wealthy also have the means to afford large quantities of food and supplies, allowing them to better maintain their physical distance from others (Rothwell, 2020; Scheiber, Schwartz, and Hsu, 2020). The ability to maintain physical distance, especially during the first year of the pandemic before widespread availability of vaccines in the U.S, is particularly important because the virus is highly contagious (CDC, 2020). At the area level, wealth remains negatively correlated with poor health and mortality, in part because of greater investment in health-promoting amenities, such as medical clinics, recreational facilities, and grocery stores, that provide greater opportunity for residents of a geographic area to remain in good health (Kawachi & Berkman, 2003).

However, wealth and race are inextricably linked. In addition to centuries of slavery, racial terrorism, redlining, and ongoing predatory lending practices, residential segregation continues to constrain the wealth that Black Americans in particular are able to amass (Du Bois, 1935; Faber, 2018; Flippen, 2004; Freund, 2007; Rugh & Massey, 2010; Taylor, 2019). Home equity is the largest asset that many Americans possess, and the homes in segregated, Black neighborhoods are consistently undervalued (Flippen, 2004; Latrice Sykes, 2003; Oliver & Shapiro, 2006; US Census Bureau, 2019). Moreover, a large body of evidence indicates that residence in Black neighborhoods is associated with poor health and high mortality (Mehra et al., 2017; Williams & Sternthal, 2010). Systemic disinvestment in Black neighborhoods, which manifests in the form of a dearth of grocery stores, medical clinics, and recreational facilities, plays an important role in this relationship between residential segregation and health (McLafferty, 1982; Moore et al., 2008; Morland et al., 2002; Whiteis, 1992; Zenk et al., 2005). These forces contribute to the disproportionately high COVID-19 mortality rate among Black Americans (American Public Media Research Lab, 2022; Centers for Disease Control, 2022).

In light of the interlocking relationships between wealth, anti-black racism, residential segregation, health, and mortality, it is important to understand how area-level wealth, residential segregation, and Black–White racial composition may be linked to COVID-19 deaths. The current paper deploys median home value at the county level as a proxy for wealth and asks two questions. First, what is the relationship between home value and COVID-19 deaths? Second, how does the relationship vary by residential segregation? Results indicate that COVID-19 mortality is 64 percent greater in the lowest wealth counties than in the wealthiest counties. At average median home value, the most segregated counties with the largest Black populations suffer 28 percent more COVID-19 deaths than similarly situated counties with low levels of residential segregation and small Black populations. This study makes two important contributions. First, it makes an empirical contribution as the first to demonstrate the linkages between wealth, residential segregation, and COVID-19 mortality rates at the area level. Second, it contributes to the literature concerned with socioeconomic status as a fundamental cause of health and disease by demonstrating that this well-established relationship depends on the level of residential segregation.

## Background and Literature Review

### Area‑Level Wealth and the Structure of COVID‑19 Vulnerability

A rich body of literature examines socioeconomic status as a fundamental cause of disease. Link and Phelan (1995) posit that socioeconomic status, including wealth, is a “fundamental cause” of disease, largely because of the persistence of the relationship between low SES and poor health over time. They argue that this relationship persists despite changes in the risk factors mediating the relationship between SES and disease throughout history.

Wealth is likely an important predictor of COVID-19 mortality because of its ability to shield individuals from vulnerability to disease. Vulnerability theory posits that susceptibility to infectious disease during a pandemic is dependent upon three processes: exposure, resistance, and recovery from the virus (Craddock, 2000; Kalipeni, 2000; McLafferty, 2010). One’s exposure to infectious disease is influenced by the social and physical environment, as well as access to resources. Resistance involves the immune system’s ability to fight disease. Important determinants of resistance include overall health, nutrition, and immunization. Recovery is the capacity to resume pre-infection activity. The process of recovery involves access to medical care and other social and economic supports (McLafferty, 2010).

I argue that wealth structures the three elements of vulnerability to infectious disease at the individual level as well as at the aggregate level, and as a result, the residents of wealthy areas likely suffer a lower COVID-19 death toll. The first way that wealth structures vulnerability to COVID-19 is by reducing exposure. At the individual level, wealth reduces exposure to COVID-19 because the wealthy are more likely to work jobs that can be performed at home, and/or possess the resources to relocate to more remote locales when COVID cases spike in their area (Gallup, 2021; Quealy, 2020; Scheiber et al., 2020). Wealthier individuals are also shielded from exposure to the virus because they are less likely to experience overcrowded living conditions, an important predictor of COVID-19 mortality (Kamis et al., 2021). Counties that have a large share of wealthy residents may therefore experience lower levels of exposure to the virus because the protections that come from individual-level wealth aggregate up to the area level. The reduced exposure that occurs as a result of many wealthy individuals residing in a geographic area may then result in lower COVID-19 mortality in the locality.

The second way that wealth may reduce vulnerability to COVID-19 is by bolstering resistance to the virus. Overall health and access to healthy food play an important role in the immune system’s ability to resist a virus such as COVID-19 (Richardson et al., 2020). At the individual level, those with greater wealth experience lower premature mortality and are less likely to suffer from chronic health issues, ranging from hypertension, to arthritis, to cognitive impairment (Adams et al., 2003; Attanasio & Hoynes, 2000). Pre-infection health, nutrition, and immunization status—central elements of the body’s capacity to resist infectious disease after exposure—are experienced at the individual level, but they are shaped in important ways by the level of wealth in a geographic area.

At the area level, extant research demonstrates that living in a wealthy area is associated with better health in a variety of domains. One important indicator of the wealth of a geographic area is median home value, because home equity is the largest asset that American households possess (US Census Bureau, 2019; Wolff, 2016). Drewnowski and colleagues (2007) find that at the zip code level, median home value is more strongly negatively correlated with the prevalence of obesity when compared to other socioeconomic indicators. The residents of wealthy areas also have greater access to grocery stores, public parks, bike lanes, and low levels of exposure to environmental toxins and crime (Kawachi & Berkman, 2003; Macintyre et al., 1993; Moore et al., 2008; Morland et al., 2002). Zahran and colleagues (2008) demonstrate that net of relevant controls, county-level median home value is linked to a greater share of the working-age population walking or biking to work. In light of the robust body of evidence that demonstrates that physical exercise and the consumption of healthy foods is central to remaining in good health (Centers for Disease Control, 2023; Harvard T.H. Chan School of Public Health, 2023; NYU Langone Health, 2023), the healthy lifestyle, and access to health-promoting resources that residence in wealthy areas affords residents may allow them to resist COVID-19 upon exposure, thus reducing mortality from the virus in the geographic area.

One important mechanism linking area-level wealth and health may be health expenditures financed by property taxes. Area-level home value is a particularly important indicator of wealth because of its relationship to property taxes. Property taxes are collected by local governments, such as cities, counties, and school districts, and they are determined in large part by the values of homes and other real property in the area (Tax Policy Center, 2013; Urban Institute, 2023b). Revenue from property taxes comprises nearly 1/3 of local revenue, and expenditures on community and public health programs, government-owned hospitals, and government payments to privately owned hospital comprise the largest share of county budgets, at 20 percent (Urban Institute, 2023a). The large property tax base in high-median-home-value localities allows for greater investment in health-promoting resources, contributing to better health for residents pre-exposure (Drewnowski et al., 2007; Mac McCullough & Leider, 2017). In light of the linkages between home value, property taxes, and health expenditures and outcomes, it is likely that residents of counties with high median home values are better able to resist COVID-19 if exposed than residents of low-median-home-value counties.

The third way that wealth may reduce vulnerability to COVID-19 is by aiding those who do fall ill in their recovery. Wealth is likely an important determinant of recovery from COVID-19, in large part because of the improved access to medical care that the wealthy enjoy. Initial evidence from New York City demonstrates that COVID-19 patients treated at medical care centers in the wealthiest parts of the city were more likely to survive than those treated in hospitals in less-affluent neighborhoods (Rosenthal et al., 2020). The wealthy also have the resources to finance medical care if they do contract a more serious case of COVID-19. In these ways, the resources that many wealthy areas afford likely reduce the probability of perishing from COVID-19.

In spite of the importance of wealth as a predictor of health and mortality, few studies deploy area-level home value as the indicator of area-level SES, and even fewer explore the linkages between housing wealth and COVID-19. One study finds that mean area property values are positively correlated with self-rated health in Paris as well as in Seattle (Jiao et al., 2016). Arcaya and colleagues (2020) find that the protective nature of housing wealth depends on increases over time in addition to the original level of area-level wealth. They demonstrate that increases in home value are linked to lower COVID-19 mortality in the highest wealth municipalities and associated with higher COVID-19 mortality in municipalities with the lowest housing value. Though this work provides important insight into the way that changes in home value are linked to COVID-19 mortality, it does not consider the ways that residential segregation may impact this relationship.

### Residential Segregation, Black–White Inequalities Across Place, and COVID‑19 Mortality

Although greater wealth at the area level may be protective against COVID-19 mortality for residents of the geographic area on average, residential segregation may complicate this relationship. Localities, such as counties and zip codes, are comprised of neighborhoods, and a large, well-established body of literature demonstrates that resources, such as the crucial health-promoting resources that impact the processes of exposure, resistance, and recovery from infectious disease during a pandemic, are not distributed equally across neighborhoods (Auchincloss et al., 2008; Kawachi & Berkman, 2003; Morrison et al., 2000; Owens et al., 2016; Wolch et al., 2011). Residential segregation across racial and ethnic lines creates inequality between neighborhoods over and above the neighborhood sorting that occurs as a result of individual preferences and income differences (Bruch & Mare, 2006; Reardon et al., 2015). Black-White segregation is particularly severe and intransigent as a result of historic and ongoing de jure and de facto policies and practices on the part of federal and state governments, real estate professionals, and individuals that invest in White places while actively withholding resources from the neighborhoods where Black households live (Agbai, Forthcoming; Korver-Glenn, 2021; Massey & Denton, 1993; Taylor, 2019).

More than a century of segregation and disinvestment in Black neighborhoods and disproportionate investment in White neighborhoods has contributed to worse health outcomes for Black residents in particular. The residents of segregated counties, metropolitan areas, and zip codes experience worse health outcomes in a variety of domains, ranging from pancreatic cancer mortality to self-rated health (Blanco et al., 2020; Cooper et al., 2001; Frankenfeld & Leslie, 2019; LaVeist, 1993; Mehra et al., 2017; Osypuk & Acevedo-Garcia, 2008; Subramanian et al., 2005; Thomas & Gaffield, 2003). In addition, residential segregation is associated with hypertension and diabetes, two major risk-factors for COVID-19 mortality (Kershaw & Pender, 2016; Kershaw et al., 2011; Richardson et al., 2020). Moreover, a growing body of work demonstrates that greater residential segregation is associated with greater COVID-19 mortality (Krieger et al., 2020; Torrats-Espinosa, 2021; Yu et al., 2021).

The relationship between residential segregation and a range of health outcomes may therefore complicate the well-established relationship between wealth and good health in a geographic area. It is possible that high levels of residential segregation in a locality concentrate the health-promoting resources in White segregated neighborhoods, severely restricting access to these resources for residents of the locality who live outside of these privileged neighborhoods. The uneven spread of health-promoting resources afforded by county-level wealth into particular neighborhoods may then impoverish overall health in the county and contribute to increased COVID-19 mortality, even in the wealthiest counties. Conversely, in counties where there are lower levels of Black-White segregation, health-promoting resources are likely spread more evenly throughout neighborhoods in the locality, increasing access for a larger share of the population. This more even spread of resources, such as recreational facilities, medical clinics, and grocery stores, may then provide a larger share of residents in the locality the resources necessary to avoid exposure, resist infection, and recover from COVID-19 if they are infected.

### The Current Study

The current study builds on the growing body of literature that examines how place contributes to COVID-19 mortality. For instance, counties and zip codes with higher levels of poverty, residential segregation, and larger Black and Latinx populations continue to suffer the highest COVID-19 mortality rates (Krieger et al., 2020; Torrats-Espinosa, 2021; Yu et al., 2021). Though this body of work provides important insight into the ways that changes in home value and residential segregation are associated with COVID-19 mortality at the aggregate level separately, it does not take into account the ways that the confluence of these factors may interact to predict COVID-19 mortality. The present study adds to this literature by examining the linkages between county-level wealth, residential segregation, and COVID-19 mortality. Specifically, I ask (1) what is the relationship between home value and COVID-19 deaths; (2) how does the relationship vary by level of residential segregation?

In light of the consistent findings from the literature concerned with socioeconomic status as a fundamental cause of health and disease, I hypothesize that wealthy areas suffer a lower death toll from COVID-19. However, in light of the large body of literature that demonstrates that health-promoting resources are spread unevenly across neighborhoods, I hypothesize that at the same wealth level, counties with high Black-White segregation experience higher COVID-19 mortality than counties with low levels of residential segregation.

## Data and Methods

### Data

Data for this analysis come from two sources. First, I deploy county-level census data from the American Community Survey (2014–2018), enumerated by the National Historical Geographic Information System (NHGIS) (Manson et al., 2019). The second data source lists total COVID-19 deaths by county and was compiled by Johns Hopkins University (Dong, Du, and Gardner, 2020). These data are compiled from the US Centers for Disease Control and Prevention (CDC) and state and local health departments throughout the country. I use data collected beginning from the first known cases in the U.S. in January of 2020, through June 24, 2021.^1^ Because this analysis deploys data from the first year of the pandemic, it provides early insights into the linkages between wealth, residential segregation, and COVID-19 mortality. The sample is restricted to metropolitan counties (n = 1,164) because the Index of Dissimilarity, a common measure of residential segregation that will be discussed in more detail below, is less reliable in rural counties (Iceland, Weinberg, and Steinmetz, 2002).^2^

### Measures

This study deploys cross-sectional data to explore the extent to which county-level wealth is linked to COVID-19 deaths.^3^ The outcome variable is the total count of COVID-19 deaths per 100,000 residents at the county level through June 24, 2021. The independent variable of interest is area-level wealth, operationalized as county-level median home value scaled to $1,000s for ease of interpretation.^4^ I select home value as the proxy for wealth in place of other measures, such as the share of homeowners in the county, because this analysis seeks to approximate aggregated wealth and its linkages to the COVID-19 death toll as closely as possible. Home equity, which is the difference between home value and the amount owed on one’s mortgage, is the largest asset that most Americans who own any wealth possess (US Census Bureau, 2019; Wolff, 2016). However, median home equity or a similar indicator is not a measure that is publicly available for the entire U.S., making median home value the next best proxy for wealth.

Because residential segregation is associated with poor health and mortality among Black residents in particular (Cooper et al., 2001; LaVeist, 1993; Mehra et al., 2017; Subramanian et al., 2005), ideal data would provide race-specific mortality at the level of the census tract. Although race-specific data are not currently publicly available at the county level for the entire nation, the Johns Hopkins and NHGIS data provide important insight into how the relationship between county-level home value and COVID-19 mortality may vary depending on residential segregation and racial composition.^5^ While the effects of wealth and residential segregation are likely more palpable at a smaller unit of analysis, such as the census tract, other recent works exploring linkages between residential segregation, racial composition, and COVID-19 outcomes also make use of the available county-level data (Kamis et al., 2021; Torrats-Espinosa, 2021; Yancy, 2020). One benefit of conducting this analysis at the county level is that few people have a hospital within the boundaries of their census tract, but there is likely a hospital present within the county. Hospital access may therefore be better captured at the county level. Though imperfect, county-level COVID-19 data provide important initial insight into the ways that wealth and residential segregation may be mutually implicated in mortality from COVID-19.

The measure of residential segregation employed in this analysis is the Index of Dissimilarity. Black-White Dissimilarity indicates the proportion of either group that would need to move out of their current neighborhoods for the two groups to be evenly distributed across census tracts in the county (Massey & Denton, 1993). This measure has important shortcomings, such as its failure to account for the spatial relationship between tracts and its inability to compare more than two groups at a time (Logan & Martinez, 2018; Massey & Denton, 1988; White, 1983).^6^ However, the use of the Dissimilarity Index allows for ease of interpretation and comparability with a wide variety of other segregation studies (Faber, 2020; LaVeist, 1993; Rugh & Massey, 2010; Subramanian et al., 2005). As a robustness check, I use the Entropy Score, rather than Dissimilarity Index, as the measure of residential segregation. Results hold with this alternate measure of residential segregation.

This analysis focuses on Black-White Dissimilarity for three reasons. First, Black Americans have suffered a disproportionately high mortality rate during the COVID-19 pandemic (Centers for Disease Control, 2021; Yancy, 2020). Second, the Black-White spatial divide is more pronounced and has been more strongly institutionalized in law and practice than the spatial, residential boundaries between other groups (Freund, 2007; Massey & Denton, 1993; Wilson, 1987). Third, the Black-White racial wealth gap is the largest in the country (Bhutta et al., 2020). For these reasons, it is important to understand the extent to which any correlation between housing wealth and COVID-19 mortality varies depending on a measure of Black-White segregation specifically.

The Dissimilarity Index can be expressed as

(1) $$D=\frac{1}{2}\sum \left|\frac{N_{1i}}{N_{1}}-\frac{N_{2i}}{N_{2}}\right|,$$

where $N_{1i}$ is the population of group 1 in census tract i of the county and $N_{2i}$ is the population of group 2 in tract i of the county. $N_{1}$ and $N_{2}$ are the total populations of groups 1 and 2 in the county, respectively. A value of D=1.0 indicates that 100% of members of one group would need to move out of their current census tracts for there to be an even distribution of that group in the county, and a value of D=0 means that both groups are evenly distributed in tracts throughout the county.

To account for the clustering of COVID-19 cases in densely populated cities like New York City and Seattle (Maxim, 2020), I control for county-level population density scaled to 1,000. I also include the share of households with 4 or more individuals to control for family transmission of the virus. Cases of COVID-19 are generally less severe among children, but particularly dangerous to those over the age of 60 (Richardson et al., 2020). I therefore include the percent of the population over age 60, and percent of households in which grandparents live with their grandchildren as controls. Following other studies of the relationship between SES and health (Ecob and Davey Smith, 1999; House et al., 1994; Meara et al., 2008; Smith & Kington, 1997), I control for logged median household income and the percent of the population with a bachelor’s degree. Because income inequality has been found to be an important predictor of an array of health outcomes at the population level, I also include the Gini coefficient (Blakely et al., 2000; McLaughlin and Stokes, 2002; Soobader & LeClere, 1999). The Gini coefficient ranges from 0 (no income inequality) to 1 (1 individual possesses all income). I also control for the share of votes cast for Donald Trump during the 2016 presidential election from the MIT Election Data and Science Lab (2018) in an effort to control for the ways that pandemic response became politicized during the early days of the pandemic in the U.S. (Lopez, 2021).

Other important covariates include the share of non-Hispanic Black and Hispanic residents, women, married-couple households, U.S. citizens, employed individuals, and insured residents at the county level (Adams et al., 2003; Brown et al., 2016; Laaksonen et al., 2009) because these demographic characteristics of the county may be related to both wealth and COVID-19 deaths. All measures are cross-sectional, and the unit of analysis is counties.

### Analytic Strategy

In the following analyses, I use the total count of COVID-19 deaths per 100,000 residents in the county as the outcome variable, and median home value as the independent variable of interest. Standard linear regression models, such as OLS, cannot properly estimate count outcome variables that are limited to positive integers. Poisson models are well suited to handle models with a positive count outcome; however, an important assumption of these models is that the mean and variance of the outcome variable are equal (Long & Freese, 2001). Because this assumption is not satisfied in the case of COVID-19 deaths, I employ negative binomial regression. This model allows for overdispersion (high levels of variability in the outcome variable) as well as count outcomes that are limited to positive integers (Long & Freese, 2001). The model can be expressed as

(2) $$COVIDDeaths_{c}=\exp\left(\beta_{0}+B_{1}HomeValue_{c}+B_{2}Dissimilarity_{c}+B_{3}X_{c}+\varepsilon_{c}\right),$$

where COVIDDeaths is the outcome variable, HomeValue is median home value, and *Dissimilarity* is Black-White dissimilarity index in county c. X represents county-level control variables, and ε is the error term. If the coefficient for HomeValue is negative, this supports the hypothesis that greater wealth is associated with fewer predicted deaths from COVID-19.

In the second step of this analysis, I create an interaction term between home value and dissimilarity to interrogate the extent to which the relationship between wealth and health holds at different levels of residential segregation. Standard errors are clustered at the state level.^7^

In the final step of the analysis, I attempt to shed light on who is perishing from COVID-19 at different levels of residential segregation in the absence of race-specific mortality rates at the county level. To do this, I plot predicted values of COVID-19 mortality by median home value for counties that fall at or below the 20th, and at or above the 80th, percentiles of Black-White dissimilarity and Black population share (hereafter, the 20th and 80th percentiles). Results hold at the 25th and 75th percentile of Black-White dissimilarity and Black population share.

## Results

### Descriptive Statistics

Table 1 provides descriptive characteristics of the counties included in the sample. An average of 177 people per 100,000 residents have died of COVID-19 per county in the United States through June of 2021, and average median home value in the sample is nearly $190,000 dollars. The average Dissimilarity Index is 0.4, meaning that 40 percent of White or Black residents would need to move out of their census tracts to another in the county for the county to be integrated.

**Fig. 1:**
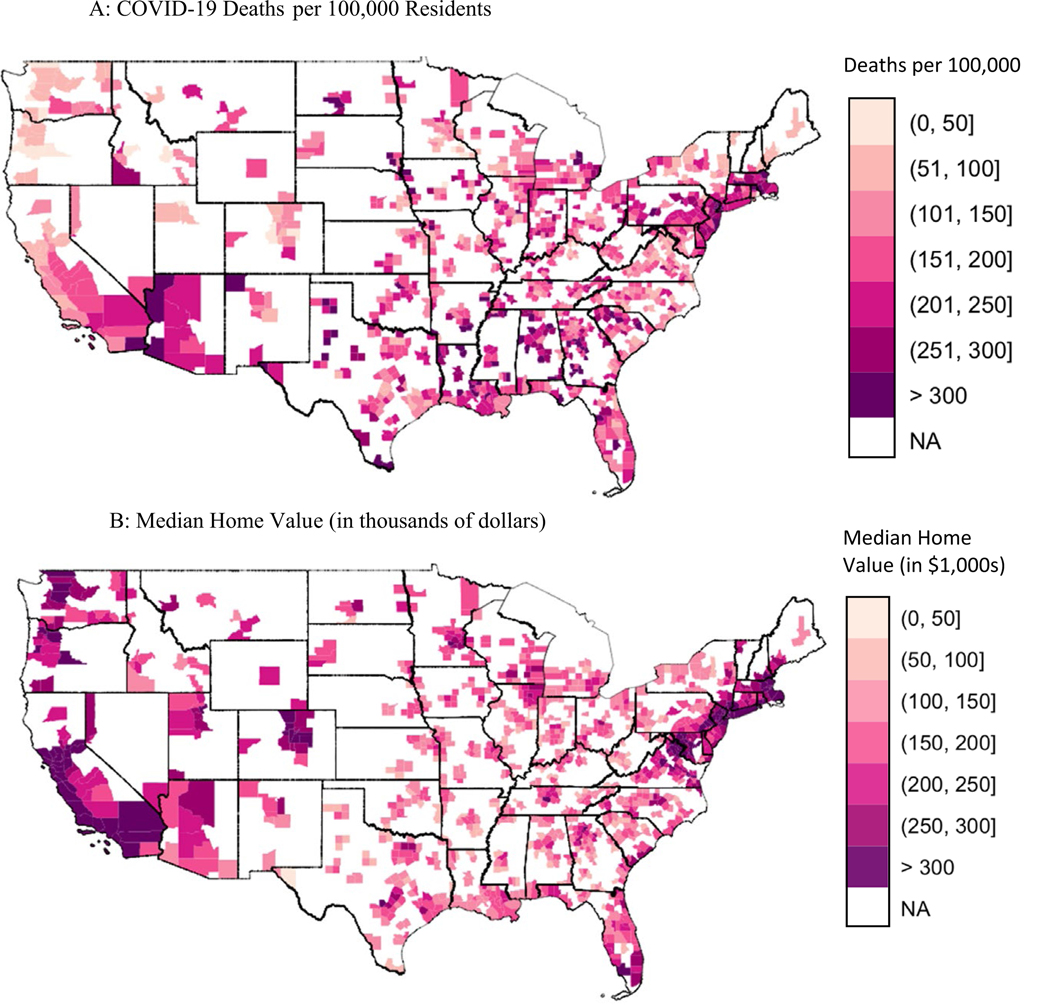
COVID-19 deaths and median home value in metropolitan counties Sources: American Community Survey data compiled by NHGIS (2014-2018), and Johns Hopkins University COVID-19 data portal (Jan 2020 through June 24, 2021).

Figure 1A maps the total count of COVID-19 deaths per 100,000 residents in metropolitan counties. Counties throughout the country have been hard-hit by the pandemic in areas including the New York City metro area, Georgia, Alabama, Texas, and Arizona. Figure 1B maps county-level median home value. Many counties with high COVID-19 mortality have low median home value, and vice versa. For instance, the counties in northern Virginia clustered near the Washington, DC area have both a relatively low number of COVID-19 deaths and high median home value, at over $300,000—nearly twice the average median home value. A similar pattern emerges in southwest Georgia, Alabama, Louisiana, Texas, and coastal California. There are important exceptions to this pattern, however. Massachusetts, Connecticut, and the New York City metro areas have both high COVID-19 mortality and high median home value. In sum, Fig. 1A and 1B provide an illustration of the spatial distribution of median home value and COVID-19 mortality, and they suggest an inverse relationship between the two at the county level.

### Linking Home Value and COVID‑19 Deaths

To evaluate the hypothesis that area-level housing wealth is negatively linked to COVID-19 deaths, this analysis employs median home value to predict COVID-19 mortality at the county level. Model 1 of Table 2 includes all county-level covariates for the full sample. In this model, each additional $1,000 in median home value is associated with a 0.2 percent decrease in expected number of COVID-19 deaths per 100,000 residents $\left(100^{*}|(1-\exp(-0.002))|\right)$. Put differently, a one standard deviation increase in median home value ($108,020) is associated with 22 percent fewer deaths $\left(100^{*}\left({\left(108,020/1000\right)}^{*}-0.002\right)\right)$ . The mean count of COVID-19 deaths per 100,000 in a county is 177, meaning that if an otherwise similar county had median home value 1 standard deviation higher, 38 fewer people would die of COVID-19 (0.22*177). Interestingly, in this model, Black-White dissimilarity and COVID-19 mortality are uncorrelated. Counties with greater population density, a larger share of residents over the age of 60, and a larger proportion of households with 4 or more people suffer more COVID-19 deaths. The Gini coefficient is strongly positive, which supports extant research that finds that residents of counties with high income inequality experience worse health (Blakely et al., 2000; McLaughlin and Stokes, 2002; Soobader & LeClere, 1999). Counties with more college-educated residents suffer fewer deaths, which aligns with extant literature that demonstrates the positive relationship between education and good health (Ross and Wu, 1995). Black and Latinx population share is also positively linked to COVID-19 mortality, supporting the growing body of literature demonstrating that these groups have been particularly hard hit by the pandemic (Centers for Disease Control, 2022; Millett et al., 2020; Torrats-Espinosa, 2021).

### Home Value and COVID‑19 Deaths at Varying Levels of Residential Segregation

**Fig. 2:**
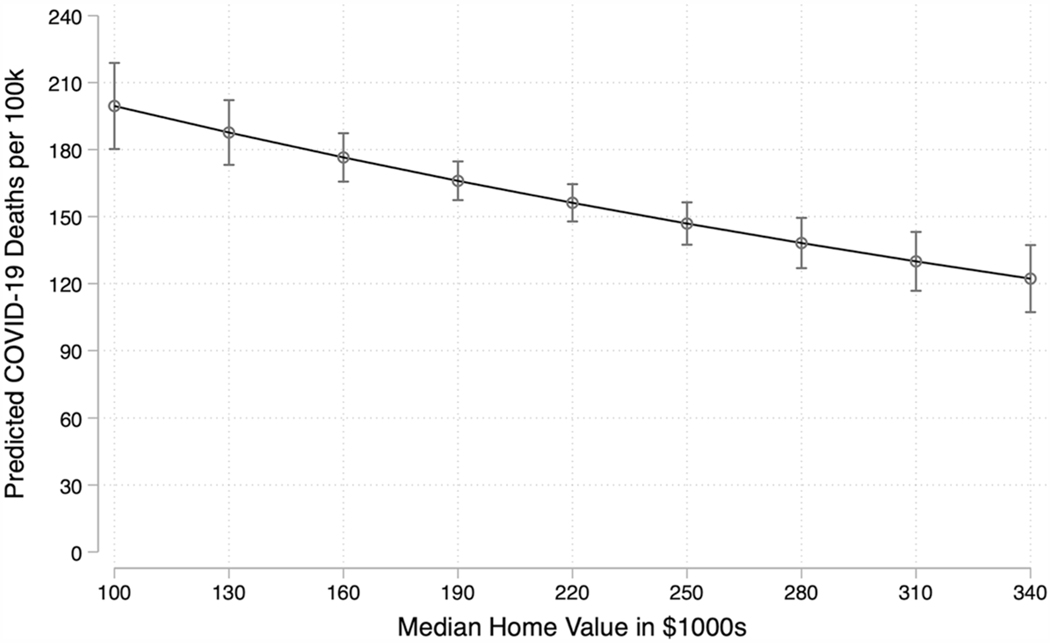
COVID-19 Mortality by Median Home Value with 95% Confidence Intervals Note: This figure plots the number of COVID-19 deaths as a function of median home value, based on Table 2 Model 2. Median home value is in thousands of dollars. All other covariates are held at their means.

**Fig. 3:**
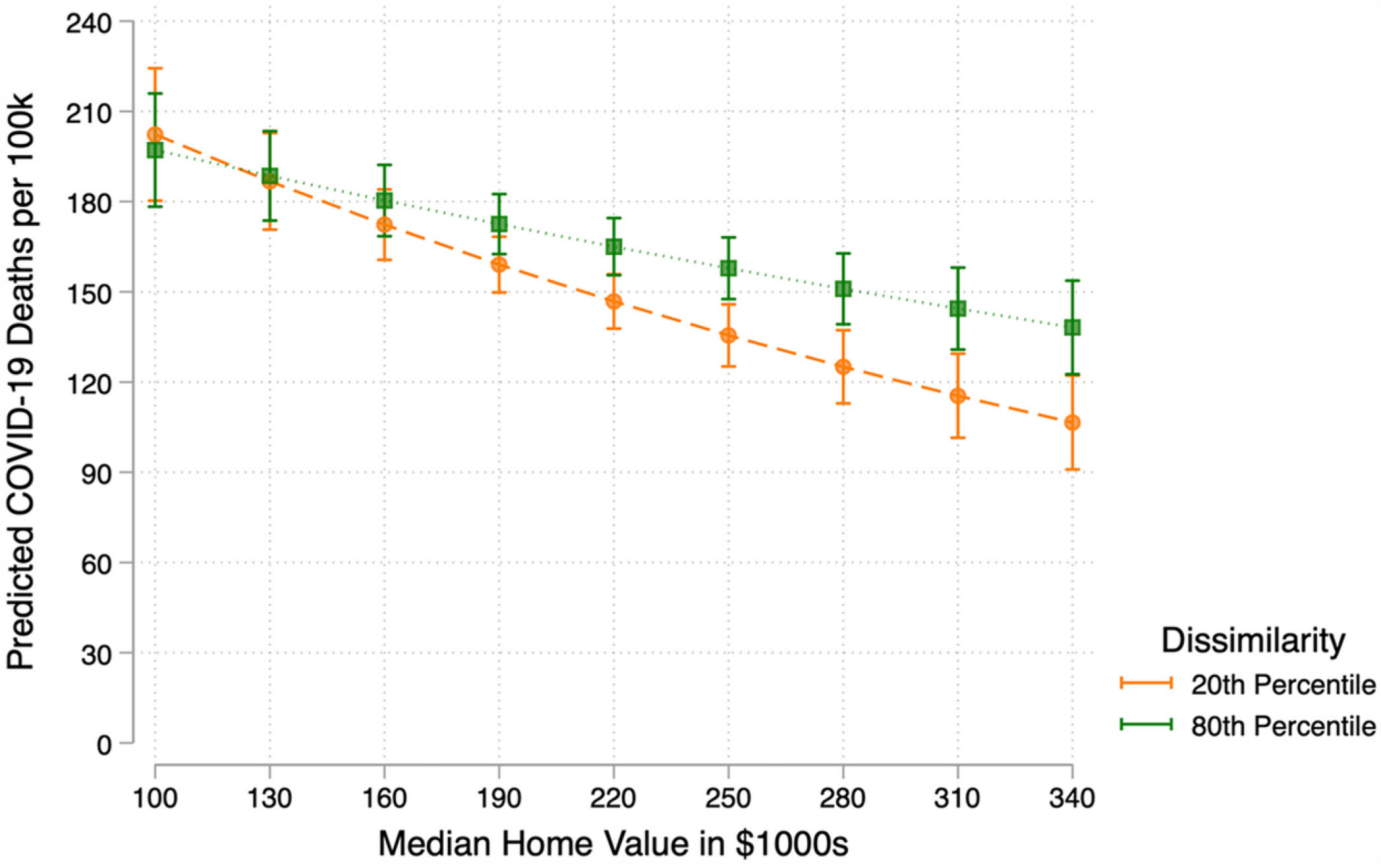
Predicted COVID-19 Mortality by Median Home Value by Percentile of Black-White Dissimilarity with 95% Confidence Intervals. (Color figure online) Note: This figure plots the number of COVID-19 deaths as a function of median home value, stratified by percentile residential segregation based on Table 2 Model 2. The 20th percentile of Black-White Dissimilarity in this sample is 0.32 and the 80th percentile is 0.55. All other covariates are held at their means.

**Fig. 4:**
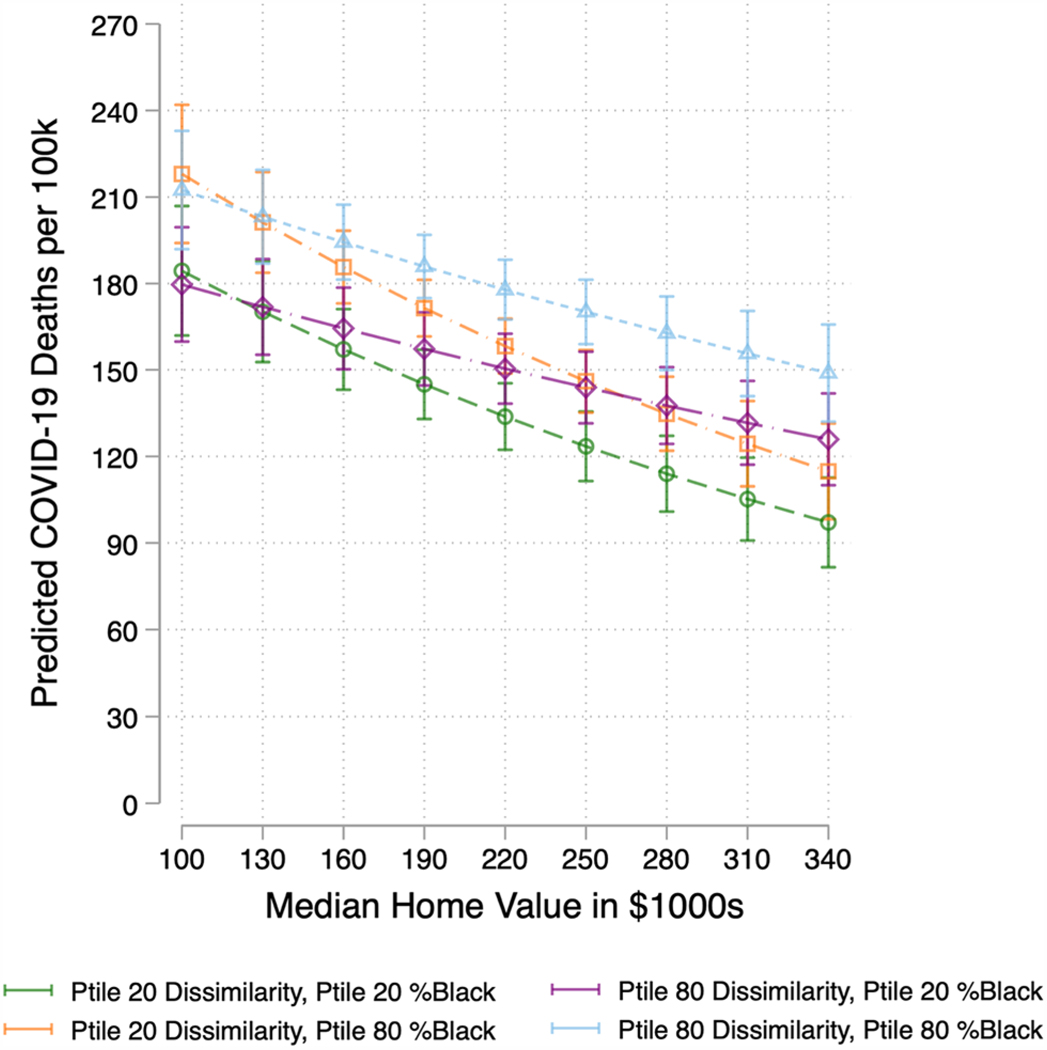
Predicted COVID-19 Mortality by Median Home Value and Percentile of Black-White Dissimilarity and Black Population Share (95% CIs). (Color figure online) Note: This figure plots the number of COVID-19 deaths as a function of median home value by level of percentile of residential segregation and Black population share based on Table 2 Model 2. The 20^th^ percentile of Black-White Dissimilarity in this sample is 0.32 and the 80^th^ percentile is 0.55. The 20^th^ and 80^th^ percentile of Black population share are 1.21 percent and 19.00 percent, respectively. All other covariates are held at their means.

This section examines the question of whether the well-established relationship between socioeconomic status and health varies depending on the level of residential segregation. Model 2 of Table 2 estimates the relationship between area-level wealth and COVID-19 mortality at different levels of residential segregation by including an interaction term between median home value and Black-White dissimilarity. The interaction between median home value and dissimilarity is positive and statistically significant. Because the interpretation of interaction terms in negative binomial regression models is not particularly intuitive, I plot predicted values in Figs. 2, 3, and 4.

Figure 2 illustrates predicted COVID-19 mortality as a function of median home value based on Table 2 Model 2, holding all other covariates, including dissimilarity, at their means. This figure demonstrates that in counties with median home value of $100,000, the predicted number of COVID-19 deaths per 100,000 residents is 200. However, counties at this level of median home value fall into the 10th percentile of the distribution of home value. A more realistic median home value in this figure is $190,000, because average median home value for the sample is $189,443. At this level, the predicted number of deaths is 166. In the wealthiest counties where median home value is $340,000, 122 people are predicted to die of the virus. Put differently, the number of predicted COVID-19 deaths per 100,000 in the lowest-wealth counties is 64 percent greater than in the wealthiest counties. In sum, results support the hypothesis that greater area-level wealth, operationalized as median home value, is negatively associated with COVID-19 deaths.

To interrogate the extent to which residential segregation complicates this relationship between area-level wealth and health, Fig. 3 plots the predicted number of COVID-19 deaths per 100,000 as a function of median home value, by level of residential segregation based on Table 2 Model 2. Where Fig. 2 plots this relationship at mean residential segregation, Fig. 3 interrogates the extent to which the negative relationship between median home value and COVID-19 mortality persists at the extremes of residential segregation. Specifically, this figure plots the relationship between wealth and COVID-19 mortality for counties that fall at or below the 20th, and at or above the 80th, percentiles of Black-White dissimilarity. This figure demonstrates that the extent to which wealth is negatively correlated with COVID-19 varies by level of residential segregation.

Throughout the high end of the distribution of median home value, it is the most segregated counties that suffer the highest mortality. For counties at the lower end of median home value that fall between $100,000 and the average median home value of 190,000, the difference in COVID-19 mortality between counties at the 20th and 80th percentile of Black-White dissimilarity is not statistically significant, though the point estimates for the 80th percentile of dissimilarity are consistently higher. At $190,000, the value closest to the average median home value, highly segregated counties suffer 172 deaths, while low-segregation counties suffer 159. For counties at the high end of median home value, however, the effect of residential segregation emerges. At median home value of $220,000, the 75th percentile of home value, COVID-19 mortality is 147 deaths at the 20th percentile of dissimilarity and 165 at the 80th percentile. At $340,000, the 90th percentile of median home value, COVID-19 mortality at the 20th and 80th percentiles of dissimilarity is 107 and 138, respectively. Put differently, at the highest levels of county median home value, the predicted number of COVID-19 deaths in highly segregated counties is significantly larger than similarly situated low-segregation counties. However, at low county median home values, the difference in COVID-19 mortality between high- and low-segregation counties is statistically indistinguishable. These results indicate that although median home value is negatively correlated with COVID-19 mortality, it is the wealthiest counties that also have low levels of residential segregation that benefit the most from area-level wealth. This finding supports the hypothesis that wealth is the most negatively associated with COVID-19 mortality in the least segregated counties, and results align with existing research that demonstrates that areas with greater residential segregation suffer higher COVID-19 mortality (Krieger et al., 2020; Torrats-Espinosa, 2021; Yu et al., 2021).

It is possible that more highly-segregated counties suffer higher COVID-19 mortality because of the unequal distribution of health-promoting resources across neighborhoods within counties (Kawachi & Berkman, 2003; Macintyre et al., 1993; McLafferty, 1982; Moore et al., 2008; Morland et al., 2002; Whiteis, 1992). The uneven spread of health-promoting resources afforded by county-level wealth into particular neighborhoods may be impoverishing overall health in the county and contributing to increased COVID-19 mortality, even in the wealthiest counties. Conversely, in counties where there are lower levels of segregation, health-promoting resources are spread more evenly throughout neighborhoods in the county, increasing access for a larger share of the population. In these less segregated counties, the well-established negative relationship between wealth and disease is the strongest, especially at high levels of median wealth. A more even spread of resources, such as recreational facilities, medical clinics, and grocery stores, may then provide a larger share of residents in the locality the resources necessary to avoid exposure, resist infection, and recover from COVID-19 if they are infected.

I now turn to the question of who is perishing in segregated counties across the wealth distribution. In the absence of race-specific COVID-19 mortality data for the entire nation at the time of this writing, I seek to shed light on the racial composition of those perishing during the pandemic. To do this, I plot the relationship between median home value and COVID-19 mortality for counties (1) at or below the 20th percentile of both dissimilarity and Black population share; (2) at or below the 20th percentile of dissimilarity and at or above 80th percentile of Black population share; (3) at or above the 80th percentile of dissimilarity and at or below the 20th percentile of Black population share; and (4) and at or above the 80th percentile of both dissimilarity and Black population share. These 4 categories provide insight into the racial composition of counties at the extremes of residential segregation to illuminate the kinds of counties where wealth is most negatively correlated with COVID-19 mortality.

Figure 4 plots the predicted number of COVID-19 deaths per 100,000 as a function of median home value, at the 20th and 80th percentiles of Black-White dissimilarity and Black population share, based on Table 2 Model 2. Several insights emerge from this figure. First, throughout the distribution of median home value, it is the counties that fall into the 80th percentile of dissimilarity and Black population share that suffer the highest COVID-19 mortality, while counties at the 20th percentile of dissimilarity and Black population share suffer the least.

At $190,000, the average median home value in this sample, highly segregated counties (80th percentile of dissimilarity) with large (80th percentile) Black populations suffer the highest death toll, at 186 deaths per 100,000. Appendix Table 3 lists median home value in counties at the 80th percentile of both dissimilarity and Black population share to ground these findings with specific examples. Highly segregated counties with large Black populations experience a large range of median home values in the sample. At the high end of median home value are counties such as Baltimore County, Maryland ($255,400); Kings County, NY, which includes the Brooklyn borough of New York City ($665,000); Cook County, IL where Chicago is located ($237,200); Fulton County, GA, which includes much of Atlanta (290,400); and Orleans Parish, Louisiana ($219,600). At the low end of the distribution of median home value in highly segregated counties with large Black populations are counties such as Alexander County, IL ($53,500); Marion County, GA ($89,500); and Jefferson County, AR ($83,800).

Counties at the 20th percentile of dissimilarity and 80th percentile of Black population share suffer the next highest COVID-19 death toll (171 deaths) at average median home value. These counties are primarily located in southern states, and they include localities such as Lowndes County, Alabama; Chattahoochee County, Georgia; and Fairfield County, South Carolina. The next highest death toll occurs in counties that are at the 80th percentile of segregation and 20th percentile of Black population share, at 157. Counties that fall into this category include locations such as Park County, Colorado; Jefferson County, Idaho; and Clay County, West Virginia. Finally, counties with the lowest levels of residential segregation and Black population share suffer the fewest deaths, at 145. Counties at the 20th percentile of both dissimilarity and Black population include locations such as Boise County, Idaho; Clear Creek County, Colorado; and Hudspeth County, Texas. In sum, at average median home value, the most segregated counties with the largest Black populations suffer 28 percent more COVID-19 deaths than similarly situated counties with low levels of residential segregation and small Black populations.

Finally, there is significant variation by Black population share within level of dissimilarity. At the 80th percentile of dissimilarity, counties with large Black populations (80th percentile) suffer higher COVID-19 mortality than counties at the same level of segregation with small Black populations. This is especially true in the middle range of median home value, between $160,000 and $250,000. This pattern also holds for counties at the 20th percentile of dissimilarity—counties at the 80th percentile of Black population share suffer significantly higher COVID-19 mortality than counties at the same level of residential segregation with small Black populations. These findings align with extant literature that demonstrate that it is not simply residential segregation, but a particularly intransigent discrimination against, and disinvestment in, Black communities that deprives these areas of the health-promoting resources that protect against a range of health issues, including COVID-19 (Auchincloss et al., 2008; Kawachi & Berkman, 2003; Korver-Glenn, 2021; Massey & Denton, 1993; Morrison et al., 2000; Taylor, 2019).

### Robustness Checks

I conduct a range of sensitivity analyses to examine the robustness of results. First, I interrogate the ways that state policy, such as stay-home orders, may have impacted the spread of COVID-19. I employ state-level data from *The New York Times* detailing the date that orders to remain at home in order to prevent the spread of COVID-19 were issued during the spring of 2020 (Mervosh, Lu, and Swales, 2020). I include the number of days from the 100th COVID-19 case in the state to the day that stay-home orders were issued as a covariate in the models. Results are robust to this addition.

In a second robustness check, I include in separate models several covariates that extant research indicates might impact COVID-19 mortality. For example, because nursing homes have been especially hard-hit by the pandemic (Yourish and Lai, 2020), I control for the number of deaths per 100,000 nursing home residents using county-level data from the Centers for Medicare and Medicaid Services (2023). The coefficient is small, positive, and statistically significant, at *p* < 0.001.^8^ The main effects of home value, dissimilarity, and the interaction between the two remain in statistical significance, direction, and magnitude. Because the inclusion of these nursing home data reduces the sample size substantially because of significant missing data in the Centers for Medicare and Medicaid Services data, I do not include this covariate in the main analysis. In a separate model, I also include average daily temperature in the county from the National Oceanic and Atmospheric Administration (2023) during the study period between January 2020 and June 2021 as a covariate because of the seasonality of COVID-19 (Wiemken et al., 2023). This coefficient is not statistically significant, while primary results hold.

Finally, I control for the share of Black residents who work in “essential” occupations because of the high rates of COVID-19 mortality in both Black communities and among essential workers (Richardson, 2020; American Public Media Research Lab, 2022). For this analysis, I deploy 2018 ACS estimates from IPUMS. Occupations deemed essential were based on media reports, as well as a report from McKinsey & Company exploring this topic (2020). Essential work for the purposes of this analysis includes service work in industries including transportation, warehousing, retail, healthcare, and construction. Results remain substantively unchanged with the addition of this covariate.

## Discussion and Conclusions

The COVID-19 pandemic has thrown the social inequalities that plague American society into sharp relief. At the outset of the COVID-19 pandemic, politicians and the news media proclaimed that the virus “does not discriminate” and that the country would struggle through the outbreak together (Hohmann, 2020; Trump, 2020; Washington Governor Jay Inslee, 2020). However, the two bodies of literature concerning the relationship between area-level wealth and mortality on one hand, and the linkages between residential segregation and health and mortality on the other suggested that this would not be the case. In this paper, I have deployed median home value at the county level as a proxy for wealth and asked two questions. First, what is the relationship between county-level median home value and COVID-19 deaths? Second, how does the relationship vary by level of residential segregation? Findings indicate that wealth is negatively correlated with COVID-19 mortality, and counties with high residential segregation and large Black populations suffer the greatest death toll. Data constraints prevent this analysis from exploring the race of individuals perishing from COVID-19 at higher rates in segregated counties with large Black populations across the wealth distribution. However, national-level and state-specific data provide consistent evidence that Black, Indigenous, and Latinx residents perished at a disproportionately high rate during the pandemic (American Public Media Research Lab, 2022; Richardson et al., 2020).

Though examining the mechanisms producing these findings is beyond the scope of this study, results align well with previous research. Results support the hypothesis that wealth structures vulnerability to COVID-19, likely because greater wealth affords localities the tax base to finance health-promoting resources, such as grocery stores, medical clinics, and recreational facilities, that reduce exposure to COVID-19, and increase resistance to, and recovery from, the virus if individuals do contract it. However, results also suggest that residential segregation so effectively clusters health-promoting resources in the most advantaged neighborhoods that residents of other neighborhoods within the county cannot benefit from them. As a result, highly segregated counties suffer a larger death toll than low-segregation counties, where resources are distributed more equally in neighborhoods throughout the county (McLafferty, 1982; Morello-Frosch & Jesdale, 2006; Whiteis, 1992; Zenk et al., 2005). The finding that living in a highly segregated, wealthy county with a large Black population is not as protective against COVID-19 mortality as living in an equally segregated county with a small Black population is consistent with extant research that finds that racism reduces the returns to socioeconomic status for Black people in particular (Pager & Shepherd, 2008; Reardon et al., 2015; Tomaskovic-Devey, Thomas, and Johnson, 2005).

Results from this study give rise to numerous questions that should be taken up in future research. First, the results presented here are descriptive and therefore cannot speak to the causal effects of home value and residential segregation on total COVID-19 deaths. Future research should explore the extent to which the linkages between wealth, residential segregation, racism, and COVID-19 deaths are causal. Second, data constraints require this analysis to be performed at the county level; however, the linkages between housing wealth, residential segregation, and COVID-19 mortality may be more visible at a smaller unit of analysis, such as the census tract. Future research should therefore make use of tract-level data for the entire nation if they become available. The current lack of race-specific COVID-19 mortality data at the county level further constrains the current study. Future work should deploy these data when they become available to more precisely interrogate the ways that place, racism, and wealth interact to contribute to COVID-19-related outcomes. Third, this analysis has explored mortality from COVID-19 as the outcome; however, severe cases of COVID-19 can have a range of long-term consequences that the scientific community is only beginning to comprehend (del Rio et al., 2020). Future work should therefore explore the extent to which wealth predicts COVID-related outcomes, such as excess death and long-lasting health issues.

This study makes two important contributions. First, it makes an empirical contribution as the first to my knowledge to demonstrate the linkages between wealth, residential segregation, and COVID-19 mortality. Second, the hypothesis that the extent to which wealth and COVID-19 mortality are linked varies depending on residential segregation is a theoretical contribution to the literature exploring socioeconomic status and health. This literature demonstrates that greater wealth at the area level is associated with better health, but this body of work does not attend to the question of the roles that residential segregation and racial composition play in this relationship. The results of the present study demonstrate that the correlation between wealth and health is highly dependent on segregation in a county. Wealth and residential segregation are inextricably linked, as residential segregation constrains the wealth that Black Americans in particular are able to amass through home equity (Flippen, 2004; Oliver & Shapiro, 2006). It is therefore important to explore the ways that housing wealth and residential segregation interact to produce and reproduce inequalities in health and mortality.

After 3 years of the COVID-19 pandemic at the time of this writing, the issues of wealth, segregation, and racism are more pressing than ever. Though the World Health Organization has declared the official end of COVID-19 as a global health emergency (World Health Organization, 2023), some unknown number of individuals are living with the long-lasting effects of the pandemic, such as the loss of loved ones and ongoing health issues as a result of infection (del Rio et al., 2020; Verdery et al., 2020). In the short term, efforts must be made to provide care to those suffering from the long-term physical and psychological effects of COVID-19. In light of the disproportionately large and detrimental impact of COVID-19 on segregated Black counties, efforts to provide ongoing physical and mental health care should be concentrated in these areas. In the longer term, the federal government, which is responsible for much of the persistent residential segregation that we see today (Agbai Forthcoming; Bruch & Mare, 2006; Freund, 2007; Massey & Denton, 1993), must invest in Black communities to the extent that it has invested in White neighborhoods over the past century in an effort to provide the kind of health-promoting resources, such as medical clinics, grocery stores, and educational institutions, that would begin to close these striking Black-White health disparities. The ongoing effects of the COVID-19 pandemic should not be ignored simply because COVID-19 is no longer designated a global health emergency by the World Health Organization. Unless decisive action is taken, it is clear that during the COVID-19 pandemic, as during non-pandemic times, health and illness, mortality and survival, will continue to be determined in large part by wealth, racism, and place.

## Figures and Tables

**Table 1:** Descriptive Characteristics of Sample

	Mean	Standard deviation

Deaths per 100,000 through June 24, 2021	176.5	85.9
Median home value	189,443	108,020
Black-White Dissimilarity Index	0.4	0.1
% Owner-occupied units	69.9	9.5
Population per square mile (in 1,000s)	628.4	2,816
County total population	228,474	494,514
% Age 60 +	22.8	4.9
% Households with grandchild(ren)	8.4	3.8
% Households with 4 + people	21.9	4.9
Gini Index	0.4	0.0
% Insured	73.1	5.0
Median household income	59,080	15,229
% Bachelor’s degree	16.7	6.1
% Hispanic	9.9	12.7
% NH Black	10.9	13.4
% U.S. Citizens	92.3	7.5
% Female	50.5	1.7
% Married	51.2	7.3
% In labor force	61.5	6.7
% Votes for Trump in County in 2016 presidential election	57.1	15.9
Total Counties	1164	

**Table 2:** The Relationship between Median Home Value and COVID-19 Mortality by Residential Segregation in Metropolitan Counties (Negative Binomial Regression)

	(1)	(2)
Variables	M1	M2

Median Home Value (scaled to $1000 s)	− 0.002*** (0.000)	− 0.004*** (0.001)
Black-White Dissimilarity Index	0.216 (0.117)	− 0.604** (0.215)
Median Home Value (scaled to $1000 s) × Black-White Dissimilarity Index		0.005*** (0.001)
% NH Black	0.010*** (0.002)	0.010*** (0.002)
Population per square mile (scaled to 1,000 s)	0.027*** (0.007)	0.013 (0.007)
% Age 60 +	5.208*** (0.798)	4.762*** (0.751)
% Households with grandchild(ren)	0.008 (0.006)	0.007 (0.006)
% Households with 4 + people	0.031** (0.010)	0.029** (0.009)
Gini Index	2.869*** (0.816)	2.376** (0.773)
% Insured	0.020*** (0.004)	0.017*** (0.004)
Logged Income	0.632 (0.327)	0.720* (0.331)
% Bachelor’s degree	− 0.017** (0.006)	− 0.015* (0.006)
% Hispanic	0.005* (0.002)	0.005* (0.002)
% U.S. Citizens	− 0.012 (0.007)	− 0.010 (0.007)
% Female	− 0.007 (0.010)	− 0.004 (0.010)
% Married	− 0.032*** (0.007)	− 0.033*** (0.006)
% In labor force	0.004 (0.006)	0.002 (0.006)
% Votes for Trump in County in 2016 presidential election	0.016*** (0.003)	0.015*** (0.003)
lnalpha	− 1.864*** (0.163)	− 1.894*** (0.163)
Constant	− 4.143 (3.159)	− 4.308 (3.166)
Total Counties	1164	1164
Number of States	50	50

Standard errors clustered to the state level in parentheses

*** *p* < 0.001,

** *p* < 0.01,

* *p* < 0.05

## Footnotes

1 In robustness checks, I use COVID-19 data through November 2020, an end date that predates the distribution of vaccines in the U.S., and results hold under this specifcation.

2 As a robustness check, I do not include the 38 counties with 0 COVID-19 deaths, and results are substantively the same.

3 Because of the overwhelmingly large number of deaths in the 5 boroughs of New York City, I perform this analysis with and without New York City. The results are substantively the same.

4 As a robustness check, I also perform the analysis with log-transformed home value, place home values into within-state deciles, and include the percent of homeowners in the models. Results that use log-transformed home value within-state deciles are substantively the same, and the coefcient for homeowners does not rise to the level of statistical signifcance.

5 Counties in the sample hold a median of 20 census tracts and an average of 52 tracts.

6 In an efort to account for the underlying racial composition of tracts within counties, I include the average number and share of Black residents in tracts within counties and use these measures as separate weights in separate models in robustness checks. I fnd that the addition of these weights produces results that are nearly identical in direction and magnitude to those of the main analysis.

7 In robustness checks, I deploy state fxed efects to estimate within state, across county diferences, and results are in the same direction and exist to a similar magnitude.

8 Results available upon request.

## Appendix

See Tables 3 and 4.

**Table 3:** Counties at the 80th percentile of Black-White dissimilarity and Black population share

State	County name	Home value	% Black	Black–white dissimilarity

Alabama	Mobile County	126,600	35.35	0.56
Alabama	Bibb County	96,500	22.08	0.55
Alabama	Jefferson County	154,400	42.54	0.64
Arkansas	Jefferson County	83,800	56.59	0.58
Florida	Broward County	243,100	27.49	0.57
Georgia	Fulton County	290,400	43.64	0.73
Georgia	Marion County	89,500	30.21	0.67
Georgia	Muscogee County	141,700	45.17	0.60
Georgia	DeKalb County	192,400	53.40	0.72
Illinois	St. Clair County	124,700	30.00	0.56
Illinois	Cook County	237,200	23.23	0.79
Illinois	Alexander County	53,500	33.21	0.61
Indiana	Lake County	143,500	23.64	0.76
Louisiana	Ouachita Parish	140,900	37.36	0.67
Louisiana	Calcasieu Parish	149,400	24.81	0.63
Louisiana	East Baton Rouge Parish	186,100	45.78	0.58
Louisiana	Caddo Parish	144,400	48.64	0.60
Louisiana	Rapides Parish	138,600	31.70	0.58
Louisiana	Orleans Parish	219,600	59.01	0.66
Maryland	Baltimore County	255,400	27.95	0.58
Maryland	Baltimore city	156,400	61.92	0.67
Massachusetts	Suffolk County	463,200	20.17	0.70
Michigan	Genesee County	104,800	19.63	0.67
Michigan	Wayne County	102,700	38.71	0.78
Mississippi	Hinds County	115,200	71.95	0.56
Missouri	St. Louis city	131,900	46.81	0.64
Missouri	Jackson County	139,000	23.41	0.61
Missouri	St. Louis County	190,100	23.98	0.71
New Jersey	Essex County	372,600	38.64	0.79
New Jersey	Union County	357,300	20.16	0.65
New Jersey	Mercer County	284,200	19.82	0.63
New York	Kings County	665,300	30.29	0.79
New York	Bronx County	382,900	29.30	0.72
North Carolina	Mecklenburg County	219,800	30.81	0.56
Ohio	Montgomery County	115,400	20.41	0.65
Ohio	Hamilton County	149,300	25.55	0.62
Ohio	Cuyahoga County	128,000	29.29	0.71
Ohio	Franklin County	165,800	21.93	0.56
Pennsylvania	Philadelphia County	156,800	41.02	0.72
Pennsylvania	Delaware County	239,600	20.92	0.69
Tennessee	Hamilton County	171,400	19.16	0.57
Tennessee	Shelby County	142,300	53.31	0.66
Texas	Dallas County	161,500	22.14	0.58
Virginia	Roanoke city	133,200	28.24	0.58
Virginia	Richmond city	220,700	47.53	0.60
Virginia	Henrico County	233,400	29.41	0.57
Wisconsin	Milwaukee County	153,600	26.10	0.76
Mean		192,938	34.09	0.65
Median		154,400	30.00	0.64
Standard Deviation		109,736	13.09	0.07
Minimum		53,500	19.16	0.55
Maximum		665,300	71.95	0.79

**Table 4:** Counties at the 20th percentile of Black-White dissimilarity and Black population share

State	County name	Home value	% Black	Black–White dissimilarity

Colorado	Gilpin County	337,400	0.25	0.00
Colorado	Clear Creek County	357,000	1.10	0.29
Colorado	Teller County	279,100	0.79	0.13
Georgia	Dawson County	215,500	0.96	0.25
Georgia	Dade County	126,700	0.44	0.28
Idaho	Boise County	199,300	0.22	0.00
Idaho	Butte County	117,600	0.15	0.00
Illinois	Woodford County	162,700	0.62	0.30
Illinois	Grundy County	191,300	1.21	0.27
Indiana	Benton County	87,900	0.57	0.25
Indiana	Harrison County	143,500	0.78	0.23
Iowa	Madison County	166,500	0.23	0.24
Kansas	Linn County	99,800	0.31	0.22
Kansas	Jefferson County	145,000	0.68	0.30
Kansas	Jackson County	132,500	0.65	0.24
Kansas	Kingman County	94,200	0.28	0.23
Kentucky	Butler County	90,100	0.27	0.31
Kentucky	Hancock County	110,300	0.93	0.31
Kentucky	McLean County	98,500	0.50	0.30
Kentucky	Pendleton County	104,900	0.80	0.24
Minnesota	Sibley County	151,000	0.85	0.13
Minnesota	Isanti County	186,000	0.44	0.31
Missouri	Caldwell County	107,900	0.52	0.06
Missouri	Polk County	125,700	0.95	0.30
Nebraska	Seward County	162,600	0.46	0.22
Nebraska	Washington County	185,800	0.84	0.19
Nebraska	Howard County	127,400	0.19	0.02
Nevada	Storey County	222,900	0.81	0.00
New York	Yates County	128,800	0.83	0.16
North Dakota	Oliver County	177,600	0.98	0.00
North Dakota	Sioux County	83,000	0.14	0.12
North Dakota	Morton County	199,800	0.44	0.29
Ohio	Fulton County	137,000	0.38	0.28
South Dakota	Union County	172,400	0.84	0.29
South Dakota	Turner County	115,000	0.33	0.07
South Dakota	McCook County	124,200	0.76	0.30
Tennessee	Macon County	112,500	0.31	0.27
Tennessee	Polk County	113,800	0.22	0.20
Texas	Armstrong County	137,700	0.37	0.00
Texas	Hudspeth County	46,000	1.20	0.00
Texas	Somervell County	191,600	0.62	0.20
Texas	Atascosa County	100,200	0.25	0.28
Utah	Juab County	203,200	0.16	0.09
Utah	Tooele County	209,700	0.50	0.31
Utah	Morgan County	359,300	0.68	0.12
Vermont	Grand Isle County	270,800	0.79	0.24
Virginia	Craig County	171,400	0.14	0.00
Virginia	Poquoson city	325,000	1.17	0.29
Washington	Columbia County	170,800	0.75	0.00
Wisconsin	Pierce County	197,500	0.43	0.31
Wisconsin	Iowa County	174,500	0.81	0.31
Mean		154,509	0.57	0.19
Median		143,500	0.52	0.24
Standard Deviation		59,645	0.30	0.11
Minimum		46,000	0.14	0.00
Maximum		359,300	1.21	0.31
